# Predictive Simulations in Preclinical Oncology to Guide the Translation of Biologics

**DOI:** 10.3389/fphar.2022.836925

**Published:** 2022-03-03

**Authors:** Shujun Dong, Ian Nessler, Anna Kopp, Baron Rubahamya, Greg M. Thurber

**Affiliations:** ^1^ Department of Chemical Engineering, University of Michigan, Ann Arbor, MI, United States; ^2^ Department of Biomedical Engineering, University of Michigan, Ann Arbor, MI, United States; ^3^ Rogel Cancer Center, University of Michigan, Ann Arbor, MI, United States

**Keywords:** antibody drug conjugate, Checkpoint inhibitors, Thiele modulus, Predictive pharmokinetics, tissue penetration

## Abstract

Preclinical *in vivo* studies form the cornerstone of drug development and translation, bridging *in vitro* experiments with first-in-human trials. However, despite the utility of animal models, translation from the bench to bedside remains difficult, particularly for biologics and agents with unique mechanisms of action. The limitations of these animal models may advance agents that are ineffective in the clinic, or worse, screen out compounds that would be successful drugs. One reason for such failure is that animal models often allow clinically intolerable doses, which can undermine translation from otherwise promising efficacy studies. Other times, tolerability makes it challenging to identify the necessary dose range for clinical testing. With the ability to predict pharmacokinetic and pharmacodynamic responses, mechanistic simulations can help advance candidates from *in vitro* to *in vivo* and clinical studies. Here, we use basic insights into drug disposition to analyze the dosing of antibody drug conjugates (ADC) and checkpoint inhibitor dosing (PD-1 and PD-L1) in the clinic. The results demonstrate how simulations can identify the most promising clinical compounds rather than the most effective *in vitro* and preclinical *in vivo* agents. Likewise, the importance of quantifying absolute target expression and antibody internalization is critical to accurately scale dosing. These predictive models are capable of simulating clinical scenarios and providing results that can be validated and updated along the entire development pipeline starting in drug discovery. Combined with experimental approaches, simulations can guide the selection of compounds at early stages that are predicted to have the highest efficacy in the clinic.

## Introduction

The design of next-generation biologics for cancer therapy has dramatically changed the drug development landscape by enabling greater control over the specificity of one (or more) molecular interaction(s) within the patient. Meanwhile, this increased complexity has made it more difficult to identify the requisite properties needed for clinical success, particularly because sophisticated therapies have multiple points of failure. Traditionally, animal experiments have been utilized for guidance on the manifold factors that impact *in vivo* and clinical efficacy. However, despite widely accepted limitations of animal results in predicting clinical outcomes, these discrepancies have become more acute with recent therapies. The result is a majority of Phase II and Phase III clinical trials ending in failure (Lowenstein and Castro, 2009).

Using antibody-drug conjugates (ADCs) to illustrate this point, different types of animal experiments are needed to gauge efficacy and toxicity. Non-human primates are often used to estimate toxicity since the targeting antibodies typically don’t cross-react with mouse antigens and expression levels are different in rodent species. Ocular toxicity, which can limit dosing from the cytotoxic small molecule payload on ADCs, may only clearly show up in rabbit models (Zhao et al., 2018). For efficacy, mouse cells are often less sensitive to ADC payloads, so human xenograft models are typically used to measure response. To examine contributions from the immune system, however, humanized or syngeneic mouse models are needed, which usually require additional engineering of the animal system. This lack of a single model to incorporate these factors exists on top of other long-standing challenges: animal species/strain differences in metabolic pathways, faster clearance in animals than humans, and immune differences between species (Bracken, 2009; Van Norman, 2019).

To bridge such gaps between *in vitro* and *in vivo* as well as animal experiments and human trials, computational approaches, such as predictive mechanistic modelling, are needed (Denayer et al., 2014). To be clear, animal data is still essential for the drug development pipeline at the present time (e.g., to predict safety/toxicity in humans). However, computational approaches are necessary to integrate this data in a quantitative manner to make informed decisions. There have been innumerable published mechanistic models which utilize the *in vitro* and/or *in vivo* results to elucidate mechanisms and to evaluate and predict efficacy in animal experiments or clinical trials. For example, some of these models focus on micro-physiological systems, such as 3-D cell culture (organoids/spheroids) (Groh et al., 2014; Hubbard et al., 2017; Cartaxo et al., 2020; Khera et al., 2021). Others include the macroscopic system, such as utilization of multi-compartment physiologically-based pharmacokinetic models (Baxter et al., 1995; Cao and Jusko, 2012; Cao et al., 2013; Groh et al., 2014). These can be expanded to combine the macroscopic features (e.g., systemic clearance and tumor uptake) with the microscopic distribution or simplified to focus on the most critical features (Cao and Jusko, 2012; Cao et al., 2013).

Sophisticated models can include many detailed mechanisms to enhance the preclinical to clinical translation of drug efficacy. For example, models for checkpoint inhibitors take experimental data including plasma clearance, organ biodistribution, tissue heterogeneity, and cellular binding to capture drug disposition (Deng et al., 2016). Li et al., 2021 started from a minimal physiologically based pharmacokinetic model by Cao et al., 2013 and applied it to pembrolizumab to predict intra-tumoral target engagement and optimal dosing (Cao et al., 2013; Li et al., 2021). For ADCs, drug processing at the cellular level plays a central role in payload release and distribution. The development of ADC models involves more complicated local metabolism/degradation features, including antibody interaction with cell surface antigens, antigen induced internalization, lysosomal degradation and release and passive diffusion of payloads (Shah et al., 2012; Cao et al., 2013; Shah et al., 2014; Cilliers et al., 2016; Singh et al., 2016). Compartmental models are sometimes inadequate to capture the heterogeneity in distribution, and ‘distributed parameter’ models are needed that capture spatial differences in drug concentration, often using penetration distance from blood vessels as a central metric (Eikenberry, 2009; Cilliers et al., 2016; Khera et al., 2018; Burton et al., 2019). More recently, hybrid agent-based models capture not only the gradients in ADC delivery, but also the heterogeneity of vessel distribution and nonuniformity of the tumor cells (e.g., heterogeneous target expression, drug sensitivity), providing more reliable prediction to clinical efficacy (Menezes et al., 2020). These models each have their strengths and limitations.

**FIGURE 1:**
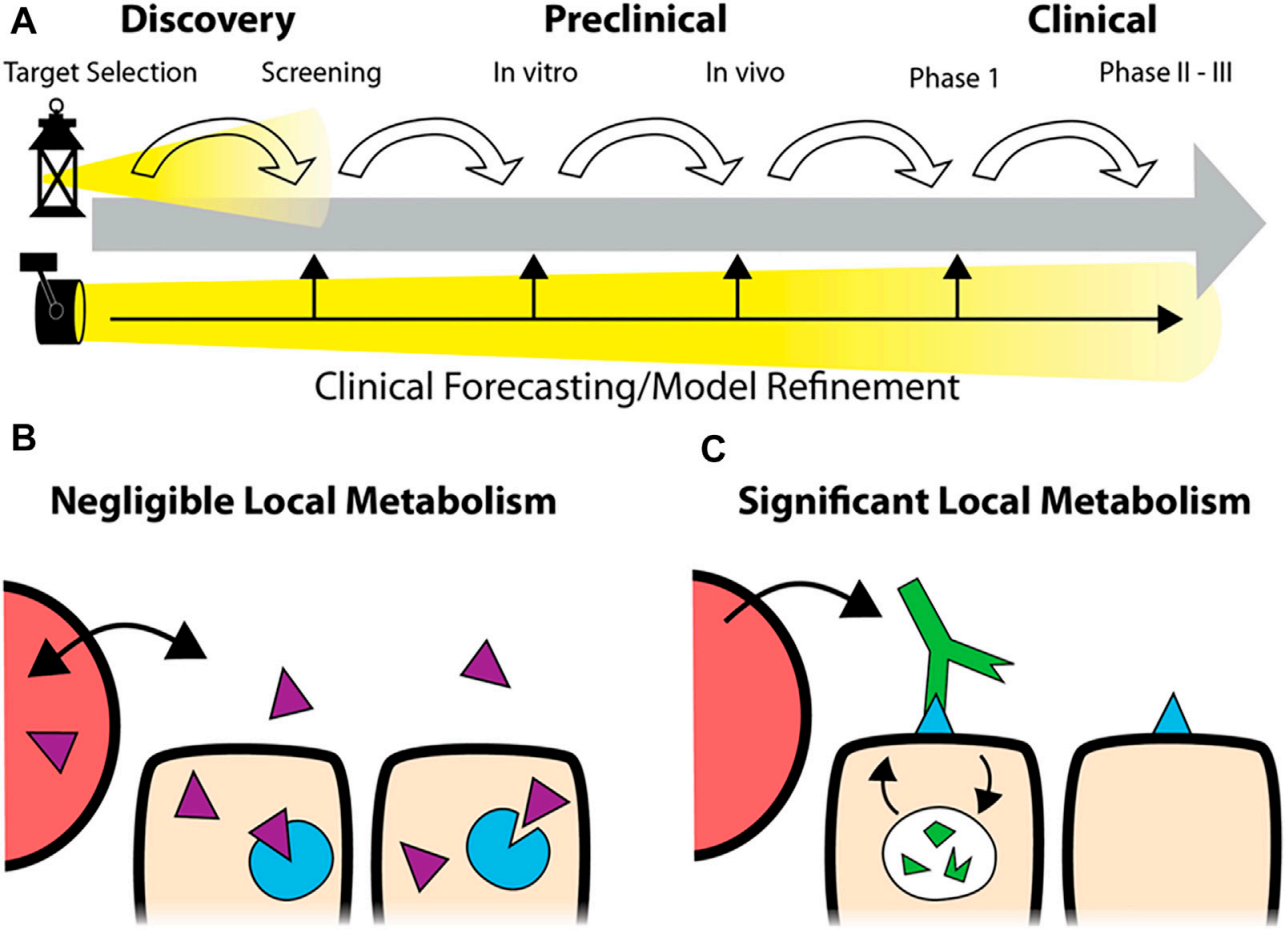
Predictive Simulations in Development. Rather than focusing on each step in the pipeline **(A)**, top, robust simulations of drug distribution can be employed at the earliest stages of development to forecast clinical application. During development, the predictions can be refined to improve the accuracy of the forecast or identify discrepancies (A, bottom). While predictive models for small molecule drugs typically assume tissue concentrations proportional to the plasma concentration due to fast distribution **(B)**, the local metabolism/degradation of biologics and slow tissue penetration require alternative approaches for accurate predictions **(C)**.

Utilization of mechanistic simulations enables insight and prediction of the processing of drugs in humans, from compartmental uptake to tissue and cellular drug distribution and efficacy. Modeling can be employed throughout the drug development pipeline, starting during the discovery phase and continuing through preclinical *in vitro* and *in vivo* testing into the clinic. These predictions can play a crucial role in avoiding poorly designed preclinical experiments and forecasting clinical trial outcomes. Importantly, these predictions should be independent from the *in vivo* experiments themselves, allowing comparison between *in silico* and experimental outcomes. The model can be refined for minor differences during development. For example, the predicted clinical dosing, initially based on archived human tissue samples, could be adjusted if the target expression is upregulated in response to treatment. In contrast, major discrepancies can signal a need to invest in further research to determine why a drug is behaving unexpectedly to avoid issues further down the pipeline, as shown in Figure 1A.

The FDA recently appealed to sponsors to determine the optimal dose instead of relying on the maximum tolerated dose (MTD) before pivotal trials. They pointed out that some MTDs lay in the over-saturating regime, producing unnecessary toxicity. Optimal dosage is often achieved when drugs are evenly distributed throughout the target compartment and saturate all targeted receptors to achieve maximum cellular response. From this perspective, drug metabolism at the cellular level, including binding, receptor internalization, recycling or degradation, combined with systemic clearance, is the determinant factor for estimating drug saturation and efficacy with most biologics.

In this work, we analyze the dosing of two classes of agents important in cancer therapy: antibody drug conjugates and PD-1/PD-L1 checkpoint inhibitors. These agents represent two cases lying far apart on the tolerability/receptor engagement scale. ADCs, with their potent payloads that can result in high toxicity, are often administered at sub-saturating doses (near the MTD) that just approach full receptor engagement at their maximum concentration (C_max_). Changes in the design impact both the MTD and receptor saturation, and agents with tolerable doses close to saturation have shown success in the clinic. In contrast, checkpoint inhibitors are generally well-tolerated antagonists which can be given at super-saturating doses. These agents are capable of maintaining full saturation even at the trough plasma concentrations (C_min_). However, without an MTD “limit,” selecting a recommended Phase II dose is difficult when the relationship between dose and efficacy is unclear (Li et al., 2021). In both cases, drug design and optimal dosing are key determinants of clinical success but challenging to identify during development. Computational tools, including simplified analysis of tumor target saturation, can provide useful insight. Some of the simplest and most predictive models can be employed early in drug development, prior to animal studies, to help guide the drug design for later stages of development. Specifically, we focus on local therapeutic degradation, which plays a central role in drug design and dosing but is often underemphasized relative to other PK metrics like plasma clearance half-life, binding affinity, and area under the curve (AUC) also used for small molecules (Figures 1B,C). Here, we utilize a previously reported dimensionless number ((Thurber et al., 2007a; Thurber et al., 2008; Wittrup et al., 2012), the Thiele modulus, to analyze the level of tumor saturation for both agents.

## Methods

### Thiele Modulus Definition

For this simplified approach, we utilize the dimensionless group, the Thiele Modulus derived for antibody pharmacokinetics (Thurber et al., 2008; Thurber and Wittrup, 2008; Wittrup et al., 2012), to describe the relative receptor saturation by accounting for tumor clearance versus delivery. While the analysis is valid for different geometries, it is defined here for a Krogh cylinder representation with the blood vessel surface area (S) to tumor volume (V): (Thurber and Dane Wittrup, 2012):

$$\frac{\text{S}}{V}\;=\;\frac{2{\text{R}}_{cap}}{{\text{R}}_{Krogh}^{2}}$$

The Thiele Modulus predicts tissue saturation by comparing the rate of vascular extravasation with endocytic consumption/degradation (Thurber et al., 2007b; Thurber et al., 2008). For high affinity antibodies (which simplifies the generalized expression for a range in antibody affinity, provided in the Supplementary Material), the expression for the Thiele Modulus is:

Φ2=ke RKrogh2([Ag]ℰ)D([Ab]1+(1/Bi))

$$Bi=\;\frac{2PR_{cap}}{D\varepsilon }$$

where $k_{e}$ is the rate constant of internalization which also represents the rate of endocytosis; $R_{Krogh}$ is the radius of the cylinder; [Ag] is the concentration of available antigen receptors (see note in supplemental data when more than 1 cell type expresses the target); [Ab] is the plasma concentration of antibody; D is the antibody interstitial diffusivity in tumor tissue; P is the antibody permeability through capillary; ε is the tumor void fraction, and $R_{cap}$ is the radius of capillary (Thurber et al., 2007b). For most antibodies, diffusion is much faster than extravasation/permeability, resulting in a small Biot number ( Bi ) (Thurber and Dane Wittrup, 2012). Therefore, the expression can be simplified to

Φ2=ke RKrogh2[Ag]2PRcap[Ab]=ke [Ag](PSV)[Ab]

Both the Biot number and Thiele modulus are dimensionless groups derived from partial differential equation models of drug distribution; therefore, the units in these expressions must cancel out. For the Thiele modulus, a faster rate of endocytosis/degradation prevents the antibodies from reaching distant tissue, resulting in limited drug penetration and unsaturated antigen receptors ( ${\text{Φ}}^{2}>1)$ . Under such circumstances, increasing the antibody dose can improve tumor uptake as well as drug distribution via increasing [Ab] . On the other hand, where saturation is achievable ( ${\text{Φ}}^{2}<1)$ , increasing the dose may maintain saturation for longer times but have limited improvement in tissue penetration.

## Results

The simplified yet predictive early-stage approach for the dosing of biologics using the Thiele modulus was applied to both ADCs and checkpoint inhibitors. The antibodies/ADCs considered here distribute as “high affinity” antibodies due to avidity and high antigen expression [where K_d_ values of 270 nM are sometimes needed to increase tissue penetration) (Rudnick et al., 2011). Likewise, lower affinity can impact internalization (Zwaagstra et al., 2019), but intrinsic receptor internalization rates are a good first approximation (Nessler et al., 2020a). A more generalized version of the Thiele modulus can describe the low affinity cases (supplemental data). ADCs are sophisticated pro-drugs that utilize a tumor targeted antibody conjugated to a potent, typically cytotoxic, payload via a cleavable or non-cleavable linker. Following intravenous administration, they circulate in the blood, are taken up in the tumor (and healthy tissue), extravasate, diffuse to their target, bind and internalize, and release their small molecule payload. The payload can then target the original cell or in the case of bystander payloads, diffuse to nearby cells. In contrast, checkpoint inhibitors block cell surface proteins which modulate immune responses and can prevent T-cells from attacking cancer cells. Instead of toxicity limitations preventing dose escalation, as is often the case for ADCs, checkpoint inhibitors don’t reach an MTD, making it difficult to determine the optimal clinical dose. Despite these major differences, the same delivery principles can be applied to both biologics to provide insight into therapeutic design and dosing.

### Thiele Modulus of Successful ADCs Are Close to 1

The doses for five FDA-approved solid tumor ADCs, mirvetuximab soravtansine, and seven checkpoint inhibitors are summarized in Table 1. Other values needed to calculate the Thiele modulus are also included in the table. The expression of PD-1/PD-L1 (tumor cells, tumor-resident T-cells and macrophages) and nectin-4 were measured to complete the table.

**TABLE 1:** A summary of package insert doses and targets of five FDA approved ADCs and seven checkpoint inhibitors.

Name	Target	Internalization half-life (hr)	Target expression (receptors/cell)	Package insert dose	C_max_ (10^−6^M)	C_trough_ (10^−6^M)	PS/V (s^−1^)
Trodelvy	Trop-2	4.06	250,000 Yuan et al. (1995), Zhang et al. (2016)	10 mg/kg D1 and D8 of 21 days cycle	1.73	∼0	6E-6 Yuan et al. (1995) Zhang et al. (2016)
Kadcyla	Her2	7 Maass et al. (2016)	1,000,000 Onsum et al. (2013)	3.6 mg/kg Q3W	0.639	0.0168	
Enhertu (IHC3+)	Her2	7	1,000,000 Onsum et al. (2013)	5.4 mg/kg Q3W	1.01	0.0787	
Enhertu (IHC2+)	Her2	7	100,000	5.4 mg/kg Q3W	1.01	0.0682	
Padcev	Nectin-4	18 Yuan et al. (1995) Zhang et al. (2016)	115,000	1.25 mg/kg D1, D8 and D15 of 28 days cycle	0.284	0.0682	
Mirvetuximab soravtansine	FR-alpha	32	1,000,000 (Forster et al. (2007)	6 mg/kg Q3W	1.09	0.0540	
Tivdak	Tissue factor (TF)	3.7 Yuan et al. (1995) Zhang et al. (2016)	112,000 Yuan et al. (1995) Zhang et al. (2016)	2 mg/kg Q3W	0.355	0.00933	
Nivolumab	PD-1	36 Lindauer et al. (2017)	5,600	240 mg Q2W	0.594	0.39 Bi et al. (2019)	6E-6 ((Yuan et al., 1995; Zhang et al., 2016) 6E-6 Yuan et al. (1995) Zhang et al. (2016)
Pembrolizumab				200 mg Q3W	0.495	0.156 Jacobs et al. (2021)	
Cemiplimab				350 mg Q3W	0.866	0.382 Kitano et al. (2021)	
Dostarlimab	PD-L1 PD-L1	35 ((Heskamp et al., 2015) 35 (Heskamp et al. (2015)	134,000 134,000	500 mg Q3W	1.24	0.278 ( (Kasherman et al. (2020)	
Atezolizumab				1200 mg Q3W	2.97	2.01 Mizugaki et al. (2016)	
Avelumab				800 mg Q2W	1.98	0.301 Doi et al. (2019)	

**FIGURE 2:**
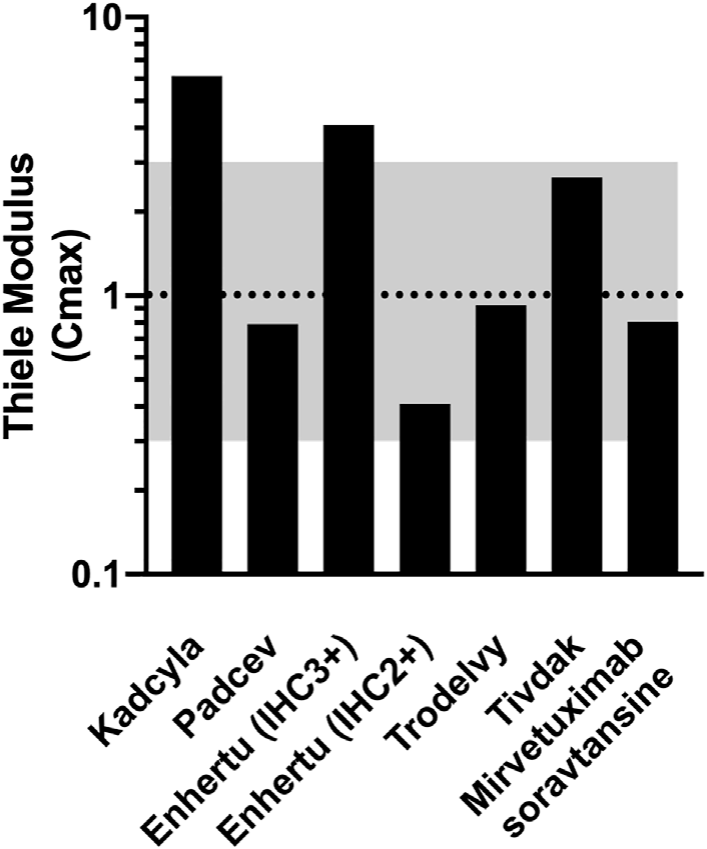
Thiele Modulus of Approved Solid Tumor ADCs and Mirvetuximab soravtansine. Values for most recent agents are close to one indicating a balance between tumor uptake and local metabolism.

The Thiele modulus was calculated from the values listed in Table 1 along with the antigen expression and internalization rate constants reported in Supplemental Table S1. The Thiele modulus of ADCs are close to one (Figure 2), indicating that endocytic consumption is not significantly faster or slower than vascular extravasation. This results in dosing that approaches saturation ( ${\text{Φ}}^{2}=1$ ) for many of the ADCs.

The Thiele modulus of Padcev and Mirvetuximab soravtansine is slightly lower than 1, indicating the dose is sufficient to overcome binding and internalization within the tumor. The antigen expression of Nectin-4 on T47D cells is lower than HER2 and Trop2, and an 18 h estimated internalization half-life (Supplementary Table S1) allows ADC molecules to quickly occupy the receptor binding sites on cell surface before they are internalized. The Thiele modulus of Mirvetuximab soravtansine is below 1 due to the low FR-internalization and recycling rate (Monteiro et al., 2020; Ponte et al., 2021). Although there’s an antibody-dependent downmodulation of TF surface expression, the Thiele modulus of Tivdak is greater than 1, consistent with heterogeneous distribution of tissue factor antibodies seen in some animal models (de Goeij et al., 2015; Koga et al., 2015). Additionally, the internalization and recycling of Tivdak is not significantly affected by binding with factor VIIa, with a half-life of 3.7 h measured by Hamik et al., 1999 (Hamik et al., 1999; Mandal et al., 2006; Breij et al., 2014; de Goeij et al., 2015). TROP2 is both highly expressed and rapidly internalized, but the high tolerability and dosing of Trodelvy helps overcome this large sink (Okajima et al., 2021). Finally, the first solid tumor ADC, Kadcyla, has a value significantly greater than one, higher than all other ADCs examined. In contrast, the higher tolerability of Enhertu allows larger dosing, resulting in a lower Thiele modulus. For patients with lower HER2 expression (IHC 2+), the Thiele modulus drops below one, balancing delivery to high and moderate expressors.

### Thiele Modulus of Checkpoint Inhibitors Are Less Than 0.1 Indicating Super-saturation

**FIGURE 3:**
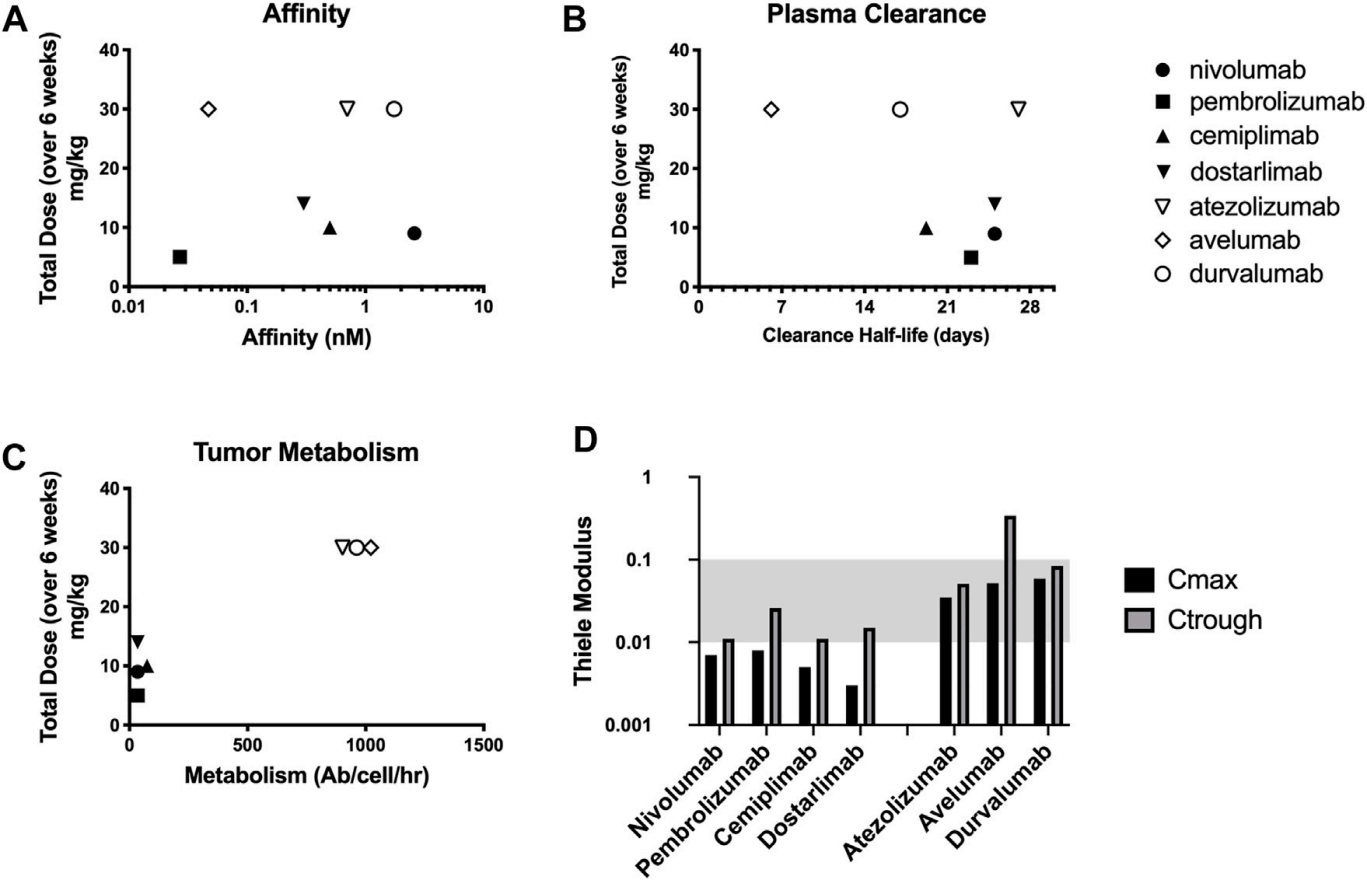
Checkpoint inhibitors vary widely in target affinity **(A)** and plasma clearance **(B)** relative to dosing. However, the doses correspond more closely with the local tumor degradation/metabolism of the drug **(C)**. Thiele Modulus of 7 different checkpoint inhibitors calculated at both C_max_ and C_trough_ showing supersaturating doses **(D)**.

The binding affinity and plasma clearance of approved checkpoint inhibitors vary widely in the clinic (Figures 3A,B). Models that assume tissue concentration is proportional to the plasma concentration (e.g., Figure 1B) indicate dosing should be related to these parameters, but there is not a correlation between approved immune checkpoint inhibitors and affinity or plasma clearance. The dosing more closely corresponds to local binding and metabolism in the tumor (Figure 3C). The Thiele modulus of PD-1 and PD-L1 inhibitors at their C_max_ and C_trough_ is shown in Figure 3D. A lower C_trough_ results in an increased Thiele modulus; however, the values are still less than 0.1 for almost all agents, indicating that PD-1 and PD-L1 proteins are saturated throughout the tumor during treatment at the FDA approved dose. With low tumor degradation due to slow checkpoint antigen internalization, the doses of PD-1 targeted antibodies are super-saturating even at the trough concentrations, i.e. ${\text{Φ}}^{2}\ll 1$ , while leaving a safety margin of 10-fold ( ${\text{Φ}}^{2}\ll 0.1$ ) for these drugs (e.g., a patient with 10-fold lower tumor vascularization would still have ${\text{Φ}}^{2}<1$ ). Due to a greater expression of PD-L1 and faster clearance, the doses of Avelumab give a Thiele modulus above 0.1 at the trough concentration but are still able to saturate the tumor.

## Discussion

By integrating data from across the drug development pipeline, computational models can help identify therapeutics that will be successful in the clinic rather than simply focused on the next step in development (Figure 1A). As more data are gathered, clinical and preclinical predictions can be refined from values based on drug structure and target properties alone (discovery phase) to incorporate *in vitro* experiments, *in vivo* results, and clinical trial data. Accurate predictions build confidence in the compound while inaccurate predictions indicate a need to better understand the system before (or while) proceeding. In fact, an inaccurate prediction can be one of the most valuable contributions of a simulation since it highlights a misunderstanding of the drug pharmacokinetics and/or response.

In addition to experimental results, some underlying fundamental principles can assist in guiding drug design and dosing. Analogous to principles such as Lipinski’s Rule of Five (Ro5) for small molecules, the Thiele modulus for biologics can provide early insight into dosing and potential delivery limitations. Values greater or less than one don’t indicate a drug will fail. However, it can motivate additional investigation into whether receptor engagement and dosing are optimally suited for clinical success. Orally available small molecule drug pharmacokinetics are usually dominated by their oral absorption (related to the Ro5) versus systemic clearance (usually centrally controlled by liver metabolism). In contrast, intravenously delivered cancer biologics are often driven by their plasma concentration (C_min_ and C_max_), vascular permeability, and local degradation (e.g., cellular expression, internalization, and degradation). The ratio of these values yields the Thiele modulus for biologics, which determines the saturation level in the tumor. Because receptor occupancy is not a static number, C_min_ and C_max_ can be used to estimate if the drug reaches all cells at the maximum concentration (important for cytotoxic delivery) or maintains full receptor occupancy at the minimum (important for antagonism of immune checkpoints). For example, an ADC with a Thiele modulus >1 may indicate the potency/DAR is too high to allow sufficient dosing to achieve tumor penetration and efficacy at a clinically tolerated dose, pointing to a reduction in potency/DAR to improve the therapeutic window. Likewise, a checkpoint inhibitor with a Thiele modulus <1 is unlikely to benefit significantly from an increase in dose since tumor target saturation is already achieved.

In fact, many of the clinical failures with ADCs point to mismatched potencies resulting in limited tissue penetration. In addition to flagging potential delivery challenges, the Thiele modulus could help identify compounds or dosing schemes that may ultimately prove more effective in the clinic based on delivery considerations, preventing them from being prematurely cut from the development pipeline. For example, the high tolerability of Trodelvy allows large dosing, improving tissue penetration into the tumor with a low Thiele modulus. However, the hydrolysable linker makes it difficult to determine an *in vitro* IC50 due to contributions from the released payload before internalization - yet it results in an effective drug (Goldenberg et al., 2015). Similarly, the *in vivo* data from Enhertu in a CT26-HER2 xenograft showed negligible efficacy due to lower sensitivity of mouse cells to the payload, yet the higher dosing and bystander payload help drive deeper tissue penetration than Kadcyla (Iwata et al., 2018). For checkpoint inhibitors, the clinical dosing of pembrolizumab was debated internally given an early signal in dose response. However, the simulations indicated receptor saturation at the lower doses, which ultimately prevailed with additional data (Patnaik et al., 2015; Elassaiss-Schaap et al., 2017).

For ADCs, the Thiele modulus varies between 0.5 and 10 with most new ADCs close to 1. The first ADC for solid tumors, Kadcyla, has the highest value. This provides an example where the analysis can raise flags but still allow successful development (similar to some successful small molecules breaking Lipinski’s Ro5). Other mechanisms, such as HER2 signaling blockade or Fc-effector function of the trastuzumab antibody, may contribute to Kadcyla’s success. Enhertu has a lower Thiele modulus due to a higher antibody dose, but it’s still greater than 1. Interestingly, Enhertu has also shown efficacy in lower expressing tumors. For these cases, its Thiele modulus for HER2+ tumors is closer to optimum. The bystander payload, where Dxd released from Enhertu can diffuse deeper into the tumor, may contribute to its efficacy at higher expression levels (IHC 3+) while saturating doses in lower expression tumors maintain effectiveness (IHC 2+). For Trodelvy and Padcev, these agents have faster plasma clearance than the other ADCs. While this may lower the exposure (e.g. AUC), a combination of high dosing and/or lower receptor expression enables them to reach most receptors for efficacy. Notably, most Thiele modulus values are not significantly less than 1, either, due to dosing limitations from the toxicity of the payload. In fact, values much less than 1 would lower the therapeutic index by increasing toxicity from the higher payload dose without a significant increase in efficacy.

Efficient internalization is critical for ADC success but can also lead to poor tissue penetration (Nessler et al., 2020b). Therefore, it’s necessary to balance the net internalization (which includes recycling) and expression level with tolerability, dosing, and potency. In fact, a lot can be gleaned from the expression and internalization rates for approved agents. For example, literature reports of the internalization rate for Nectin-4 indicate an ∼18 h half-life with 10^5^ receptors/cell, which is one of the lowest rates of uptake for the 5 solid tumor approved ADCs (M-Rabet et al., 2017). Correspondingly, Padcev has one of the highest potencies in terms of combined payload and DAR (DAR 4, MMAE) among these ADCs. On the other extreme of these approvals, Trodelvy has both higher expression (between 10^5^ and 10^6^ Trop2/cell) and much faster internalization (4.06 h half-life) (Cardillo et al., 2015). It also has the lowest potency payload (SN38) which is only partially compensated by the higher DAR (DAR 8)—hence greater tolerability and higher dosing. Finally, Kadcyla and Enhertu are in-between, with higher expression than Nectin 4 but slower internalization than Trop2 (Austin et al., 2004). Both utilize moderate payloads and DARs, with Dxd and DM1 having greater potency than SN38 but less than MMAE. Comparing these two HER2-targeting constructs, the lower potency of Dxd is only partially compensated by higher DAR (DAR 8 for Dxd vs DAR 3.5 for DM1 on Kadcyla), resulting in greater tolerability/dosing of Enhertu. Overall, the higher expression and faster internalized targets are paired with lower potency ADCs (a product of payload potency and DAR), which enables higher dosing for better tissue penetration. This balance between intrinsic payload potency/DAR, expression, internalization, and dose results in most approved ADCs with a Thiele modulus close to 1.

The situation is very different for checkpoint inhibitors, but the same fundamental principles apply. The Thiele modulus of PD-1 and PD-L1 inhibitors at their maximum concentrations are all below 0.01 and 0.1, respectively. Even at lower trough concentrations, all except the fastest clearing agents are still below 0.1, indicating supersaturation of the receptor. In most early phase development trials, data reported on pharmacodynamic properties of PD-1 and PD-L1 inhibitors suggest that a MTD dosing scheme supersaturates receptors. In fact, the MTD is often not reached with checkpoint inhibitors in clinical trials (Brahmer et al., 2010; Herbst et al., 2014; Powles et al., 2014; Sehgal et al., 2020). Studies by Topalian et al., 2012 and Agrawal et al., 2016 have shown that with a dose of nivolumab at 0.1–0.3 mg/kg, which is about ten-fold lower than the approved dose (Table 1), maximal occupancy of PD-1 receptors can be achieved (Topalian et al., 2012; Agrawal et al., 2016). Data collected by Song et al., 2015 also indicated that soluble PD-L1 receptors were fully saturated in a majority of patients treated with durvalumab at 0.3 mg/kg every 2 weeks (Song et al., 2015). A similar result has been observed by Antonia et al., 2019 where complete soluble PD-L1 suppression is achieved (Antonia et al., 2019).

Supersaturating doses may be acceptable for agents that are well-tolerated, but the higher doses of checkpoint inhibitors do come at a cost. These doses not only increase the expense of treatment but can exacerbate anti-drug antibody (ADA) responses, particularly for agents that are designed to increase immune responses (Hock et al., 2015; Davda et al., 2019; Enrico et al., 2020). The situation raises some important drug development questions. Current dosing provides a 10X or greater ‘safety margin’ (i.e. dosing above tumor saturation) according to this analysis. The result is consistent with a relatively flat dose response curve at these levels (e.g., atezolizumab (Boswell et al., 2019)). What cost (in terms of ADA risk and material price for all patients) is acceptable for additional benefit in a subset of patients (such as those with poorly vascularized tumors that require a higher dose)?

Ironically, higher dosing can not only increase the risk of ADA but simultaneously overcome the same problem. For example, atezolizumab has been reported to induce ADA responses in 39.1% of the safety-evaluable patients, but the large dose appears to also prevent an impact on efficacy (Davda et al., 2019). Systemic exposure of atezolizumab is lower in ADA-positive patients due to enhanced clearance, but there was no significant impact on efficacy. Therefore, even with a good understanding of receptor occupancy, outstanding questions remain. Is it better to utilize lower doses to prevent ADA responses or higher doses to overcome the problem? Is an ADA response impairing drug efficacy and/or an indication that the immune system has been activated? The best answer will depend on the specific drug, but analysis of dosing and receptor occupancy with these trade-offs are important to consider.

Another uncommon feature of checkpoint inhibitors is tumor versus healthy tissue target saturation. Typically, antibodies saturate receptors in healthy tissue at lower doses than the tumor (enabling strategies such as preblocking healthy tissue) (Boswell et al., 2019). This occurs due to healthy tissue having a combination of higher and more uniform vascularization, better convection and lymphatic drainage (versus impaired convection from elevated interstitial pressure in tumors), and often lower target expression relative to tumors (Jain et al., 2007; Zhang et al., 2016). A collection of permeability and S/V values for healthy tissues has been published by Zhang et al. (Zhang et al., 2016) and can be used to predict healthy tissue saturation. However, compartmental/PBPK models are better suited for analyzing healthy tissue uptake (Mager and Jusko, 2001). Healthy tissue saturation often coincides with achieving linear plasma pharmacokinetics, where the dose is high enough to saturate receptors (reducing targeted mediated drug disposition, TMDD) such that the clearance rate becomes constant and plasma concentrations are proportional to dose (Patnaik et al., 2015). Linear pharmacokinetic profiles have been observed during treatment with most checkpoint inhibitors at 1 mg/kg (0.3 mg/kg for pembrolizumab (Patnaik et al., 2015; Elassaiss-Schaap et al., 2017); 0.1 mg/kg for nivolumab (Patnaik et al., 2015; Elassaiss-Schaap et al., 2017); 1 mg/kg for atezolizumab (Stroh et al., 2017; Sehgal et al., 2020), cemiplimab (Yang et al., 2021), and avelumab (Heery et al., 2015) and 3 mg/kg for the PD-L1 inhibitor durvalumab (Patnaik et al., 2015; Elassaiss-Schaap et al., 2017), implying that the approved doses saturate normal tissues. In contrast, predictions indicate tumor saturation occurs at much lower doses primarily due to lower target expression (the product of immune cell count and receptors/cell) in tumors where cancer cells often outnumber tumor infiltrating lymphocytes. Interestingly, this enables the possibility for tumor specific inhibition, where a lower dose saturates lymphocytes in the tumor without saturating the receptor in tissues with much denser receptor concentration (e.g., lymph nodes).

Finally, receptor expression is not static. Upon treatment with anti-PD-1 and anti-PD-L1 therapies, Vilain et al., 2017 showed an infiltration of PD-1+ T-cells in tumor, as well as upregulation of tumoral PD-L1 and macrophage PD-L1 of responders (Vilain et al., 2017). There’s also a large amount of literature addressing the regulation of PD-L1 expression in cancer cells mediated by cytokines or transcriptional pathways (Tremblay-LeMay et al., 2018). A relatively high dose of checkpoint inhibitors can compensate for upregulation of antigens and prolong the duration of an effective treatment. This is an example of where literature values and *in vitro* estimates of target expression from the discovery phase can be updated with preclinical *in vivo* data or *ex vivo* clinical data to further refine the clinical predictions. Quantitative measurements of animal and clinical expression are essential. With absolute receptor expression levels (rather than semi-quantitative metrics like IHC or H-scores), computational models can offer advantages over animal models by tailoring the results to the clinic. For example, the level of vascularization has a significant impact on PS/V and therefore delivery. Computational models can vary the PS/V values to those seen in the clinic (or even a particular tumor type or patient) rather than a given animal model. Additionally, by varying the S/V, this analysis can also be applied to healthy tissue for the prediction of therapeutic window.

As the biologics used to treat cancer increase in complexity, it is important to develop computational methods alongside animal experiments to better predict clinical outcomes. The fact that animal experiments can give opposite results depending on their design (e.g., a high or low DAR is more effective depending on the dose used) means that the preclinical outcomes are not necessarily providing fundamental insight into how the drug will behave in the clinic but rather how the drug behaves in that specific experiment (Nessler et al., 2021). Computational predictions, grounded in experimental data, can help translate how these results will manifest in patients for better decision-making during development.

In conclusion, the success of biologics in cancer therapy not only relies on the biology of the target but equally on forecasting the dosing and drug design for clinical efficacy. Mechanistic computational models can predict how drugs will translate from the discovery to *in vitro*, *in vivo*, and clinical stages. This includes simple and robust metrics, such as the Thiele modulus derived from computational models, that can provide insight into how currently successful drugs are behaving and guide the design and dosing of future therapeutics.

## Data Availability

The original contributions presented in the study are included in the article/Supplementary Material, further inquiries can be directed to the corresponding author.
