# Supplementary material for: Predictive Simulations in Preclinical Oncology to Guide the Translation of Biologics
Source: Front Pharmacol. 2022 Mar 3;13:836925. doi: 10.3389/fphar.2022.836925 (PMC8927291; doi:10.3389/fphar.2022.836925)
Supplement: Supplementary file 1 [file DataSheet1.PDF]

Predictive Simulations in Preclinical Oncology to Guide the Translation of Biologics

Shujun Dong, Ian Nessler, Anna Kopp, Baron Rubahamya, and Greg M. Thurber

Supplementary Materials and Methods

Generalized Thiele Modulus Expression

The Thiele modulus is the dimensionless ratio of the capillary extravasation rate and the endocytic consumption rate, describing tumor penetration.[1] A generalized Thiele modulus expression is obtained by substituting parameters for antibody delivery, such as metabolism rate (internalization rate) and vascular extravasation rate. Specifically, the saturable binding kinetics can account for low affinity antibodies where the K_d impacts tissue penetration[2, 3]

$$\Phi^2 = \frac{k_e\, R_{Krogh}^2 \left({[Ag]}/{\varepsilon}\right)}{D\left(\frac{2PR_{cap}[Ab]}{D\varepsilon} + K_d\right)}$$

where k_e is the rate constant of internalization which also represents the rate of endocytosis; R_{Krogh} is the radius of the cylinder; $[Ag]$ is the concentration of available antigen receptors (see note in supplemental data when more than one cell type expresses the target); $[Ab]$ is the plasma concentration of antibody; D is the antibody interstitial diffusivity in tumor tissue; P is the antibody permeability through capillary; ε is the tumor void fraction; R_{cap} is the radius of capillary, and K_d is the antibody binding affinity. This generalized Thiele modulus expression accounts for the effect from both low and high affinity antibodies. However, "high affinity" antibodies are mainly considered in this work because most bivalent antibodies behave as high affinity due to avidity in high expressing system.[4] Given the antibody doses in Table 1, the K_d of the antibody would generally need to be greater than 10 nM to have a significant impact on tissue distribution. The tumor vascular permeability depends on the size of the molecule, and a value for an IgG-sized protein is used here. Schmidt et al. presented a fitted two-pore model of the capillary wall to correlate the permeability with the molecule radius.[5]

Antibodies and Cell Lines

T47D, NCI-N87, CT26, and 4T1 cells were purchased from ATCC. Antibodies included anti-Nectin-4-AF647 (337516 clone, R&D Systems), CD45-FITC (30-F11 clone, BD Biosciences), CD3-PE (17A2 clone, BD Biosciences), F4/80-PE (6F12, BD Biosciences) and hRS7 from

Immunomedics. AlexaFluor 488 from Thermo Fisher Scientific were conjugated to the antibodies as previously described.[6-8] Briefly, NHS ester dyes were reacted with antibodies (>2mg/mL) in aqueous solution with 10% sodium bicarbonate (v/v) for 3 hours under room temperature. Labeled antibodies were purified using Biogel P-6 (Bio-RAD) in a Costar Spin-X column by centrifuging at 3500x g for 1 min.

Measurement of Nectin-4 expression
To determine the Nectin-4 expression, T47D cells were removed from the plate by incubating with 0.05% Trypsin-EDTA solution for 5 minutes. The suspension was washed twice with PBS to remove excess trypsin and incubated with 40nM of anti-Nectin-4-AF647 for 40 minutes on ice, followed by 2X washes with PBS. The suspension was then analyzed using an Attune Acoustic Focusing Cytometer. Quantitative fluorescent beads purchased from Bangs Laboratories (Quantum Simply Cellular anti-human IgG) were used to convert fluorescent intensity units from flow experiments to antibody binding capacities.

Measurement of Trop2 internalization
The Trop2 internalization rate was measured as previously described.[9] NCI-N87 cells were seeded into 24-well plates at a density of 3×10^5cells per well and allowed to adhere for 12hrs. After washing cells once with PBS, 300μL of Alexa-488 conjugated hRS7 antibodies at a concentration of 40nM in media was added to each well and incubated either at 37°C or on ice. Non-specific uptake of antibodies was measured by pre-blocking the antigen receptors using unlabeled antibodies at 37°C for 15 minutes followed by incubation with 40nM of labeled antibodies in the pre-blocking solution. At each time point, NCI-N87 cells were chilled on ice first and washed twice with PBS, followed by incubating with 300μL of 0.05% Trypsin-EDTA for 15 minutes at 37°C. Cell suspensions were transferred in Eppendorf tubes, washed twice with PBS to remove excess trypsin and set on ice till the time for flow cytometry analysis. Half of the cells were resuspended in trypan blue solution to quench surface fluorophore.

PD-1/PD-L1 measurements
All animal experiments were conducted under the approval of the Institutional Animal Care and Use Committee (IACUC) at the University of Michigan and followed the National Institutes of Health guidelines for animal welfare. For in vivo PD-1/PD-L1 receptor expression measurements, 6-8 week old Balb/c mice were inoculated in a single flank with syngeneic mouse cell lines (either $1x10^5$ 4T1 or $5x10^5$ CT26 cells). The volume of these tumors was monitored until an average tumor volume of $300mm^3$ was measured. At this point, mice were euthanized and the tumor resected. The tumor was then digested into a single-cell suspension with the Miltenyi Biotech tumor dissociation kit and passed through a 40 μm filter to remove clumped cells. The suspension was washed in PBS-2%BSA and then incubated with either CD45-FITC, CD3-PE, F4/80-PE, PD1-AF647, and/or PDL1-AF647 for 30 minutes on ice. Afterwards, Cells were pelleted and supernatant removed, followed by 2 washes with PBS-2%BSA. The final pellet was resuspended in PBS and analyzed using an Attune Acoustic Focusing Cytometer. The fluorescent PD-1 and PD-L1 antibodies were quantified for fluorescence using Quantum Simply Cellular anti-Rat IgG beads to convert from fluorescence to number of antibodies per cell.

Table 1. Summary of target expression and internalization half-life values from literature.

Target	Receptor expression<br>(Cell line: Expression)	Internalization half-life<br>(Cell line: half-life)
Her2	BT-474: 2.7×10^6/cell<br>NCI-N87: 3.25×10^6 /cell<br>SK-BR-3: 3.55×10^6/cell [10]	BT-474: 6.3hr<br>NCI-N87:13.6hr<br>SK-BR-3: 7.7hr [10]<br>Multiple-dose mouse xenograft: 5.8hr [1]
Trop2	HCC1806: 1.4×10^5 /cell<br>NCI-N87: 2.0×10^5 /cell<br>BxPC-3: 3.0×10^5 /cell<br>Caov-3: 2.4×10^5 /cell[11]	NCI-N87: 4.06hrs*
FR-α	SW620: 6.9×10^5 /cell<br>IGROV: 8.3×10^5 /cell[12]<br>10^6 /cell<br>SKOV3: 10^5 /cell [13]<br>KB: 4×10^6 /cell<br>OV90: 4×10^4 /cell[14]	HeLa: ~2×10^5 molecules/hr<br>KB: ~3×10^5 molecules/hr<br>IGROV: ~1×10^5 molecules/hr[15]<br>1.5×10^5 molecules in the first hour[12]<br>OV90: 32hr[14]
Nectin-4	T47D: 115,000/cell*	MDA-MB-231: 60% decrease of surface receptor was observed in 24hrs[16]

* - values from *in vitro* measurements.

Table 2. Summary of PD-1/PD-L1 expressions on different cell types and fraction of cells in tumor.

Tumor		Tumor cells	T-cells	Macrophages	Average expression
CT26	Percentage of TME (±SD%)	87.4±1.9	7.34±0.95	2.84±0.45	
	PD-1/cell (± SEM)	1,220±190*	24,500±3,000	95,300±8,400	5,600
	PD-L1/cell (± SEM)	44,800±5,600	662,000±46,000	1,640,000±204,000	134,000
4T1	Percentage of TME (±SD%)	64.1±6.8	14.0±2.9	3.60±2	
	PD-1/cell (± SEM)	1,380±370*	9,600±1,300	43,000±8,900	3,800
	PD-L1/cell (± SEM)	8,220±750	362,000±6,200	720,000±43,000	82,000

* - values likely represent non-specific interactions

The averaged expressions of checkpoint inhibitors are calculated by summing up the product of target expression and fraction of cells in tumor over all cell types.

$$Average\ expression = \sum_{cell\ types} \{(fraction\ of\ cells\ in\ tumor)(targets/cell)\}$$

References:

[1] G. M. Thurber, S. C. Zajic, and K. D. Wittrup, "Theoretic Criteria for Antibody Penetration into Solid Tumors and Micrometastases," *Journal of Nuclear Medicine,* vol. 48, no. 6, p. 995, 2007, doi: 10.2967/jnumed.106.037069.

[2] G. M. Thurber, M. M. Schmidt, and K. D. Wittrup, "Antibody tumor penetration: Transport opposed by systemic and antigen-mediated clearance," *Advanced Drug Delivery Reviews,* vol. 60, no. 12, pp. 1421-1434, 2008/09/15/ 2008, doi: https://doi.org/10.1016/j.addr.2008.04.012.

[3] K. D. Wittrup, G. M. Thurber, M. M. Schmidt, and J. J. Rhoden, "Practical theoretic guidance for the design of tumor-targeting agents," (in eng), *Methods in enzymology,* vol. 503, pp. 255-268, 2012, doi: 10.1016/B978-0-12-396962-0.00010-0.

[4] S. I. Rudnick *et al.*, "Influence of Affinity and Antigen Internalization on the Uptake and Penetration of Anti-HER2 Antibodies in Solid Tumors," *Cancer Research,* vol. 71, no. 6, pp. 2250-2250, 2011/3// 2011, doi: 10.1158/0008-5472.CAN-10-2277.

[5] M. M. Schmidt and K. D. Wittrup, "A modeling analysis of the effects of molecular size and binding affinity on tumor targeting," *Molecular Cancer Therapeutics,* vol. 8, no. 10, p. 2861, 2009, doi: 10.1158/1535-7163.MCT-09-0195.

[6] C. Cilliers, J. Liao, L. Atangcho, and G. M. Thurber, "Residualization rates of near-infrared dyes for the rational design of molecular imaging agents," *Molecular imaging and biology,* vol. 17, no. 6, pp. 757-762, 2015.

[7] C. Cilliers, B. Menezes, I. Nessler, J. Linderman, and G. M. Thurber, "Improved Tumor Penetration and Single-Cell Targeting of Antibody–Drug Conjugates Increases Anticancer Efficacy and Host Survival," *Cancer Research,* vol. 78, no. 3, p. 758, 2018, doi: 10.1158/0008-5472.CAN-17-1638.

[8] I. Nessler, C. Cilliers, and G. M. Thurber, "Practical Guide for Quantification of In Vivo Degradation Rates for Therapeutic Proteins with Single-Cell Resolution Using Fluorescence Ratio Imaging," *Pharmaceutics,* vol. 12, no. 2, 2020, doi: 10.3390/pharmaceutics12020132.

[9] M. M. Schmidt, G. M. Thurber, and K. D. Wittrup, "Kinetics of anti-carcinoembryonic antigen antibody internalization: effects of affinity, bivalency, and stability," (in eng), *Cancer immunology, immunotherapy : CII,* vol. 57, no. 12, pp. 1879-1890, 2008, doi: 10.1007/s00262-008-0518-1.

[10] K. F. Maass, C. Kulkarni, A. M. Betts, and K. D. Wittrup, "Determination of Cellular Processing Rates for a Trastuzumab-Maytansinoid Antibody-Drug Conjugate (ADC) Highlights Key Parameters for ADC Design," (in eng), *The AAPS journal,* vol. 18, no. 3, pp. 635-646, 2016, doi: 10.1208/s12248-016-9892-3.

[11] D. Okajima *et al.*, "Datopotamab Deruxtecan, a Novel TROP2-directed Antibody–drug Conjugate, Demonstrates Potent Antitumor Activity by Efficient Drug Delivery to Tumor Cells," *Molecular Cancer Therapeutics,* 2021, doi: 10.1158/1535-7163.MCT-21-0206.

[12] P. M. Smith-Jones *et al.*, "Preclinical radioimmunotargeting of folate receptor alpha using the monoclonal antibody conjugate DOTA-MORAb-003," (in eng), *Nuclear medicine and biology,* vol. 35, no. 3, pp. 343-351, 2008, doi: 10.1016/j.nucmedbio.2007.12.008.

[13] S. Miotti *et al.*, "Growth of ovarian-carcinoma cell lines at physiological folate concentration: effect on folate-binding protein expression in vitro and in vivo," *International journal of cancer,* vol. 63, no. 3, pp. 395-401, 1995.

[14] J. F. Ponte *et al.*, "Antibody Co-Administration Can Improve Systemic and Local Distribution of Antibody–Drug Conjugates to Increase In Vivo Efficacy," *Molecular Cancer Therapeutics,* vol. 20, no. 1, p. 203, 2021, doi: 10.1158/1535-7163.MCT-20-0451.

[15] C. M. Paulos, J. A. Reddy, C. P. Leamon, M. J. Turk, and P. S. Low, "Ligand Binding and Kinetics of Folate Receptor Recycling in Vivo: Impact on Receptor-Mediated Drug Delivery," *Molecular Pharmacology,* vol. 66, no. 6, p. 1406, 2004, doi: 10.1124/mol.104.003723.

[16] M. M-Rabet *et al.*, "Nectin-4: a new prognostic biomarker for efficient therapeutic targeting of primary and metastatic triple-negative breast cancer," *Annals of Oncology,* vol. 28, no. 4, pp. 769-776, 2017, doi: https://doi.org/10.1093/annonc/mdw678.
